# Serum microcystin-LR levels and risk of gestational diabetes mellitus: A Chinese nested case-control study

**DOI:** 10.3389/fendo.2022.1047866

**Published:** 2023-01-04

**Authors:** Ying Lin, Rongjing An, Chunli Wu, Huixia Liu, Jing Deng, Hongzhuan Tan, Lizhang Chen, Mengshi Chen, Shujuan Ma

**Affiliations:** ^1^ Department of Epidemiology and Health Statistics, Xiangya School of Public Health, Central South University, Changsha, China; ^2^ Hunan Provincial Key Laboratory of Clinical Epidemiology, Central South University, Changsha, China; ^3^ Reproductive and Genetic Hospital of CITIC-Xiangya, Clinical Research Center For Reproduction and Genetics In Hunan Province, Changsha, China

**Keywords:** gestational diabetes mellitus (GDM), microcystin-LR (MC-LR), nested case-control study, endocrine disrupting chemicals (EDCs), risk

## Abstract

**Background:**

Previous experimental studies have reported an association between microcystin-LR (MC-LR) and glucose homeostasis, but whether exposure to MC-LR is a risk factor for the pathogenesis of gestational diabetes mellitus (GDM) requires further epidemiological study. This study aims to explore the effects of MC-LR on GDM.

**Methods:**

A prospective nested case-control study was performed in the Hunan Provincial Maternal and Child Health Hospital (HPMCHH) in South China. A total of 119 patients with GDM and 238 controls were enrolled in the study. The two independent samples t-test, or chi-square test was used to compare the difference between the GDM group and the non-GDM group. Binary logistic regression was used to obtain odds ratios (ORs) by controlling for confounders.

**Results:**

The cumulative incidence of GDM in our sample was 13.7%. The detection rate of MC-LR in the GDM group were significantly higher than those in the control group (44.2% vs. 29.4%; *p*=0.007). Our results show that an elevated serum MC-LR level in the first trimester of pregnancy was related to an increased risk of GDM (OR: 1.924; 95% CI: 1.092-3.391; *p*<0.05). When stratified by age, educational level, parity, and passive smoking, significantly relationships were observed among those aged >30 years, lower income, higher education, none passive smoking, and more likely to be multiparous.

**Conclusions:**

Our data reveals that serum MC-LR level in the first trimester is independently associated with GDM.

## 1 Introduction

Gestational diabetes mellitus (GDM) is defined as glucose intolerance with onset or first recognition during pregnancy (1). It is one of the most common complications of pregnancy. The prevalence of GDM has been rapidly increasing. Zhu Y et al. reported that the prevalence of GDM ranges from 1.7% to 11.6% by reviewing the data available from the past decade (2). GDM has been linked to various adverse health outcomes in pregnant women and fetuses, including hypertensive disorders complicating pregnancy (HDCP), polyhydramnios, premature labor, and macrosomia (3–5). In addition, women with a history of GDM during pregnancy appear to have a nearly 10-fold increased risk of developing type 2 diabetes mellitus later in life (6). Therefore, to reduce the incidence of GDM, it is imperative to identify the risk factors associated with its development.

Microcystins (MCs) are a family of cyclic heptapeptide endotoxins released during cyanobacterial blooms. The increase in water eutrophication has led to the excessive proliferation of MCs in water bodies, and there is emerging concern about the potential adverse effects of MCs on human health. Among the 270 different structural variants of MCs, microcystin-LR (MC-LR) is the most common and toxic variant (7, 8). MC-LR is found mainly in water bodies, which could accumulate in aquatic wildlife and to be transferred to higher trophic levels with the risk of animal and human. MC-LR can easily gain access to the human body through the consumption of polluted drinking water, skin contact during recreation, or food intake (9, 10). MCs are commonly detected in lake reservoir type water bodies. MCs have been detected in Taihu Lake, Chaohu Lake, Erhai Lake and Dianchi Lake in China. In recent years, the frequency of blue algae outbreaks in Dongting Lake in Hunan Province has also increased. The level of MCs in water and health risks need to be concerned. Various studies have shown that MC-LR is associated with developmental toxicity in the fetus (11), impairment of renal function (12), and hepatocellular carcinoma (13). Notably, MC-LR acts similarly to endocrine disrupting chemicals (EDCs) (11) that have been associated with dysregulated glucose metabolism in GDM and type 2 diabetes (14–16). However, there is lack of population-based evidence on the association between MC-LR and GDM.

Several animal and cell experiments have demonstrated that MC-LR may be associated with diabetes (17–20). MC-LR can induce a lipid metabolic disorder (17). Lipid metabolic abnormalities are closely related to incidences of GDM, and therefore MC-LR may be involved in the pathogenesis of GDM (21). Moreover, previous studies have demonstrated that MCs can accumulate in pancreatic islet cells, which may exert toxic effects on islet cell function (22–26). A progressive decline in pancreatic islet cell function is characteristic of diabetes (27, 28). Additionally, previous studies showed that the toxic reaction caused by MC-LR was mainly achieved by inhibiting protein phosphatase 1 (PP1) and protein phosphatase 2A (PP2A) (11). Some studies have found that PP2A is also related to metabolic diseases (29, 30). PP2A can affect the survival of pancreatic islet cells and the ability of pancreatic islet cells to secrete insulin (31, 32). Taken together, we hypothesize that exposure to MC-LR is associated with an increased risk of developing GDM.

In order to verify the experimental results of the association between MC-LR and glucose homeostasis, we conducted a nested case-control study to investigate whether exposure to MC-LR in the first trimester of pregnancy is associated with an increased risk for developing GDM in Hunan, China.

## 2 Methods

### 2.1 Study population

The nested case-control study was based on data from a cohort study established in the Hunan Provincial Maternal and Child Health Hospital (HPMCHH) in South China (ChiCTR1900020652). Pregnant women in their first trimester (10-14 weeks) were recruited and followed up for 42 days postpartum. The specific inclusion criteria were the following: (i) singleton pregnancy and natural conception; (ii) diabetes-free at recruitment, without diseases that might affect microbiome composition or glucose metabolism, such as pre-pregnancy diabetes, thyroid disorders, hypertension, inflammatory bowel disease or cardiovascular diseases; (iii) has not received any antibiotic treatment throughout the current pregnancy; (iv) no acute infection 2 weeks before sample collection; and (v) planned to complete regular obstetric examinations and delivery at the current hospital. For each patient with GDM, two controls were randomly selected from patients in the cohort who did not develop GDM. This study was approved by the Hunan Provincial Maternal and Child Health Hospital Institutional Review Board and is in accordance with the principles of the Declaration of Helsinki.

### 2.2 Data collection

Participants completed interviewer-administered and validated questionnaires on baseline characteristics and lifestyle, including information on age, gravidity, parity, education, socioeconomic status, family history of diabetes, and anthropometric measures. Gestational weight gain was calculated by subtracting pre-pregnancy weight from maternal weight at birth. All of the questionnaires were completed by trained research staff in accordance with the instructions.

### 2.3 Diagnosis of GDM

GDM is diagnosed at 24-28 gestational weeks of pregnancy using established criteria from the International Association of Diabetes and Pregnancy Study Groups (IADPSG) based on the results of a standard 2 h, 75 g oral glucose tolerance test (OGTT). Pregnant women were diagnosed with GDM if glucose levels were elevated with one or more of the following present: fasting≥5.1 mmol/L, 1 h≥10.0 mmol/L, and 2 h≥8.5 mmol/L (33).

### 2.4 MC-LR measurement

Blood samples (3-5 mL) were collected during early pregnancy (10-14 weeks of gestation) by certified nurses in the morning following a 10-hour overnight fast. All of the samples were stored at 4°C immediately after collection and transported back to the laboratory on ice within 8 hours. Blood samples were centrifuged at 3500 rpm for 15 min, and the serum collected from each sample was divided into three equal parts and stored at -80°C until tested.

MC-LR levels in serum were measured using direct competitive ELISA kits (Beacon Analytical Systems Inc., USA). A total of 50ul of enzyme label was added to each well in the microplate. 50ul of standard solution, negative control solution, and sample were added to the corresponding microwells, followed by 50ul of antibody solution added to each well. The solution was shaken in the wells, applied to the film, and incubated for 30 minutes. The solution was then removed into the water tank and the plate was washed 5 times. 100ul of substrate solution was added to each well. After incubation for 30 minutes, 100ul of stop solution was added to each well. Absorbance at 450nm was measured using a spectrometer. The absorbance of the standard solution was used to plot the calibration curve. The levels of MC-LR in the serum specimens were plotted according to their absorbance. The limit of MC-LR was 0.1 ng/ml.

### 2.5 Covariates

Age was divided into 2 groups: <30 and ≥30. Body weight (kg) and height (m) were measured in light clothes and without shoes. Body mass index (BMI) was calculated by dividing the weight (kg) by the square of the height (m). Participants were classified as underweight/normal weight (BMI < 24.0 kg/m^2^), overweight (≥24 kg/m^2^), or obese (BMI ≥ 30.0 kg/m^2^). Pre-pregnancy BMI was categorized into two groups: <24 and ≥24 kg/m^2^ (34, 35). Educational level was divided into two groups: high school or below and junior college or above. Monthly income levels (RMB) were divided into two groups: <10000 and ≥10000. MC-LR levels were stratified into low- MC-LR levels (<0.1 ng/ml) and high-MC-LR levels (≥0.1 ng/ml) according to the limit of detection of serum MC-LR levels.

### 2.6 Statistical analysis

According to the data type, two independent samples t-test, or chi-square test was used to analyze the differences between the GDM group and control group. Binary logistic regression models were used to obtain odds ratios (ORs) and their 95% confidence intervals (95% CIs) in univariable and multivariable analyses. Multivariable analysis was adjusted for age, pre-pregnancy BMI, weight gain during pregnancy, education level, occupation, household income, family history of diabetes, parity, and passive smoking. A *p*-value < 0.05 was considered statistically significant.

## 3 Results

### 3.1 Characteristics of the study population

During the study period, a total of 870 women were included in the cohort. Of these women, 119 developed GDM. The cumulative incidence of GDM was 13.7%. The basic characteristics of the cases (n=119) and controls (n=238) are summarized in **Table 1**. Compared with women without GDM, women with GDM were older, had a higher pre-pregnancy BMI, had more weight gain during pregnancy, were more likely to be multiparous, and had higher MC-LR levels during their first trimester care visit (*p*<0.05). There were no statistically significant differences between the two groups in terms of education level, occupation, household income, family history of diabetes, or passive smoking.

**Table 1:** General characteristic of GDM cases (N=119) and controls (N=238).

Variables	Non-GDM N=238	GDM N=119	*P*-value^a^
Mean ± SD			
**Age (years)**	28.41 ± 4.37	31.63 ± 4.65	**<0.001**
**Pre-pregnancy BMI (kg/m^2^)**	19.97 ± 2.10	22.09 ± 3.08	**<0.001**
**Weight Gain During Pregnancy**	15.44 ± 4.2	13.07 ± 5.4	**<0.001**
*Number (%)*			*P*-value^b^
**Educational level**			**0.829**
High school and below	34 (14.3)	16 (13.4)	
Junior college and above	204 (85.7)	103 (86.6)	
**Occupation**			**0.210**
Employed	205 (86.1)	108 (90.8)	
Unemployed	33 (13.9)	11 (9.2)	
**Household income, RMB/month**			**0.111**
<10000	145 (60.9)	62 (52.1)	
≥10000	93 (39.1)	57 (47.9)	
**Family history of diabetes**			**0.192**
No	215 (90.3)	102 (85.7)	
Yes	23 (9.7)	17 (14.3)	
**Parity**			**0.001**
0	158 (66.4)	58 (48.7)	
≥1	80 (33.6)	61 (51.3)	
**Passive smoking**			**0.133**
No	203 (85.3)	94 (79.0)	
Yes	35 (14.7)	25 (21.0)	
**MC-LR**			**0.007**
Low (<0.1 ng/ml)	185 (70.6)	53 (55.8)	
High (0.10-0.23 ng/ml)	77 (29.4)	42 (44.2)	

a from Student’s t-test.

b from chi-square test.

### 3.2 MC-LR levels between subgroups

**Table S1** lists the concentration distribution of serum MC-LR, with the detection rates being 33.3%. Distribution of serum MC-LR between different characteristics of pregnant women is displayed in **Table S2**. MC-LR levels were similar among these different pregnant characteristics.

### 3.3 Associations between MC-LR and GDM risk

**Table 2** presents the results of the binary logistic regression analysis for GDM in relation to MC-LR by different models. Pregnant women with higher MC-LR levels during their first trimester showed a significantly higher risk of developing GDM (OR: 1.924; 95%CI: 1.092-3.391, *p*=0.010) than those with lower MC-LR levels before and after adjusting for maternal age, pre-pregnancy BMI, weight gain during pregnancy, education level, occupation, household income, family history of diabetes, and parity.

**Table 2:** Logistic regression analysis for GDM in relation to maternal MC-LR.

Variable	Non-GDM	GDM	ORc (95%CI)	OR^a^ (95%CI)
MC-LR				
Low (<0.10 ng/ml)	185 (70.6)	53 (55.8)	1	1
High (0.10-0.23 ng/ml)	185 (70.6)	53 (55.8)	1.904 (1.173-3.090)*	1.924 (1.092-3.391)*

a Adjusted for age, pre-pregnancy BMI, weight gain during pregnancy, education level, occupation, household income, family history of diabetes, parity, and passive smoking.

*P < 0.05.

### 3.4 Stratified analysis on the association between MC-LR and GDM risk

**Figure 1:**
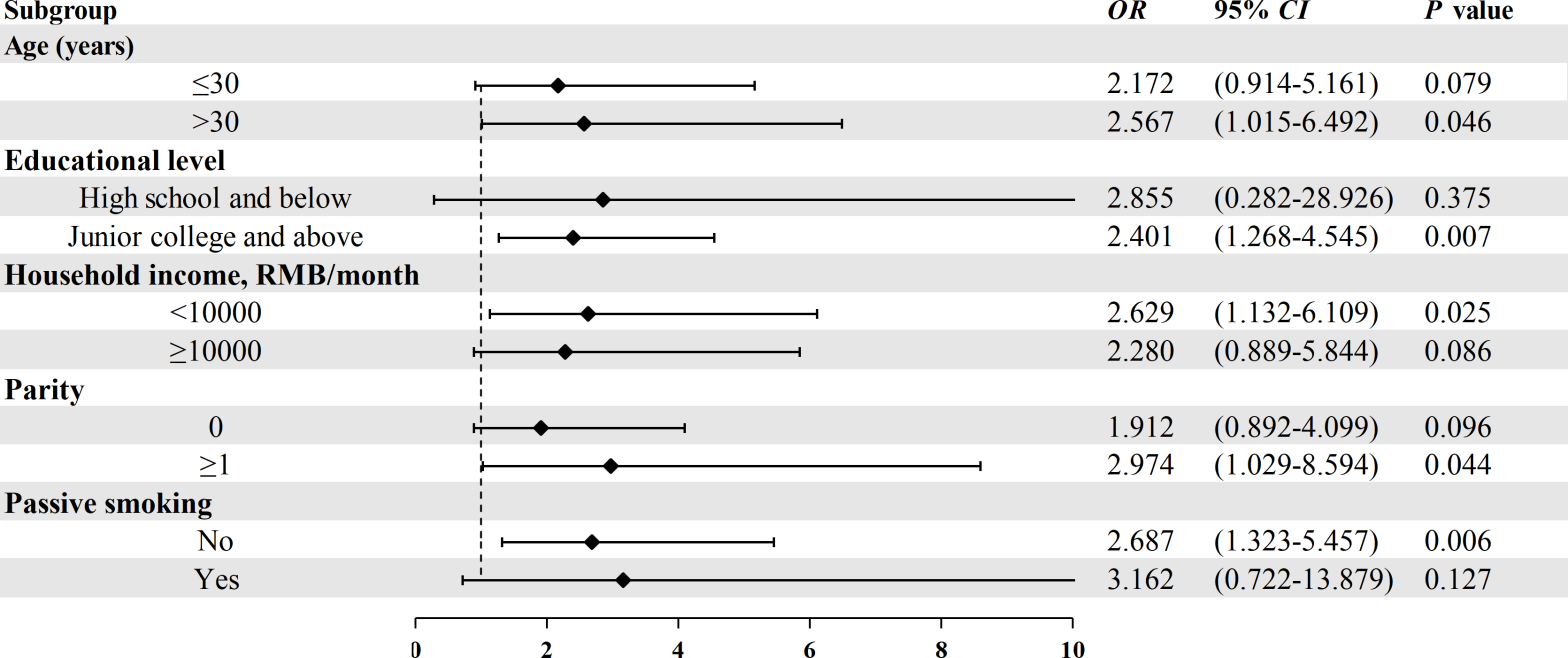
Stratified analysis on the association between serum MC-LR and the risk of GDM. Model adjusted for age, pre-pregnancy BMI, weight gain during pregnancy, education level, occupation, household income, family history of diabetes, parity, and passive smoking.

The association between MC-LR and GDM risk stratified by age, educational level, parity, and passive smoking was displayed in **Figure 1**. Because the sample size is too small after stratification according to BMI, Occupation and diabetes family history, there is no stratification analysis for these variables. After stratification, MC-LR was still associated with the GDM risk; however, the strength of the associations slightly differed in some subgroups. Stronger significantly relationships were observed among those aged >30 years, lower income, higher education, none passive smoking, and more likely to be multiparous.

## 4 Discussion

The cumulative incidence of GDM was 13.7% in our study, which was similar to the reported incidence of GDM (14.8%) from a meta-analysis of 25 studies in mainland China (36). In addition, we demonstrated that serum MC-LR levels in early pregnancy were significantly and positively associated with GDM.

The serum MC-LR levels in our study were lower than the levels reported from studies in other areas in China, namely Chongqing (37, 38), Anhui (10), and Hubei (39). In China, populations living in rural areas or near lakes are more likely to be exposed to MCs, compared to most of our research participants who were living in cities.

Our study shows that pregnant women with increased serum MC-LR in the first trimester had a higher risk of developing GDM, which was consistent with the previous laboratory studies (17–20, 40). However, epidemiological studies on the association between the exposure to MC-LR and diabetes or GDM are scarce. Ecological research from Lake Taihu showed significantly higher incidence of type 2 diabetes mellitus (T2DM) than that from other areas of China; this may be related to the Microcystis bloom in the area (41). Yanyan Zhao et al. reported (19) the potential adverse effect of exposure to MC on pancreatic islet cell function in human populations and found that a subset of the surveyed participants had lower blood insulin levels and impaired plasma glucose regulation. These findings support the hypothesis that exposure to MC-LR can impair pancreatic islet cell function. In contrast to the previous cross-sectional studies, our study used a prospective study design to investigate the association between serum MC-LR levels in early pregnancy and the risk of developing GDM. Our results further support the hypothesis that exposure to MC-LR may play an important role in the pathogenesis of GDM.

Therefore, the increasingly serious nitrogen and phosphorus pollution, cyanobacteria bloom and algal toxin pollution of China’s water resources have directly threatened the safety of urban residents’ drinking water and aquatic products. It can be seen that the treatment of water pollution is imminent

The potential mechanisms involved in the association between MC-LR and GDM are unclear. One possible explanation may be that exposure to MC-LR induces an inflammatory response (42–44), and inflammation is considered a risk factor for developing GDM (45, 46). Additionally, several studies have reported that exposure to MC-LR increases oxidative stress in the pancreatic islets (47, 48), which may result in β-cell dysfunction. MC-LR activates the NF-κB signaling pathway to up-regulate iNOS expression and induce cell apoptosis, which may partially inhibit pancreatic islet β-cell proliferation (18, 49). Meanwhile, chronic exposure to MC-LR may impair glucose tolerance and induce insulin resistance (17). Since insulin resistance and β-cell dysfunction are two key events in the pathogenesis of GDM, it is plausible that exposure to MC-LR may predict GDM. These findings help explain the association between MC-LR and GDM.

As mentioned above, MC-LR enters the body mainly due to drinking contaminated drinking water and skin contact. The increasingly serious nitrogen and phosphorus pollution, cyanobacteria bloom and algal toxin pollution of water resources have directly threatened the safety of urban residents’ drinking water and aquatic products, so it is urgent to control water pollution.

The potential limitations of the current study should be considered. To begin with, we only measured the serum levels of MC-LR, without accounting for the potential serum levels of other MCs. However, MC-LR has been known to the most abundant and toxic form, hence our choice of measurement. Additionally, although our results showed that exposure to MC-LR may increase the risk of developing GDM, further experimental evidence is needed to better understand the molecular mechanisms of how MC-LR is involved in the pathogenesis of GDM. Moreover, although we have collected as many risk factors of GDM as possible into the model, there may still be risk factors that have not been considered. Finally, as the study population of this study is mainly from Hunan Province, and due to the differences in living habits and diets in different regions, multi-center studies with larger sample sizes are required to validate our results.

## 5 Conclusion

Serum MC-LR levels in the first trimester were positively associated with a greater risk of developing GDM. Our research can provide reference for improving people’s awareness of the necessity of controlling pollution of water. Further studies are needed to validate these findings and investigate the feasibility of water pollution intervention on reducing the level of exposure to MC-LR to prevent the developing GDM during pregnancy.

## Data availability statement

The raw data supporting the conclusions of this article will be made available by the authors, without undue reservation.

## Ethics statement

The studies involving human participants were reviewed and approved by Medical Ethics Committee of Hunan Maternal and Child Health Hospital. The patients/participants provided their written informed consent to participate in this study. Written informed consent was obtained from the individual(s) for the publication of any potentially identifiable images or data included in this article.

## Author contributions

MC, SM, and YL designed the study. YL, RA, CW, and HL recruited participants, collected basic data and samples. MC, YL, and SM analyzed the data. JD, MC, LC, HT, and YL contributed to discussion and reviewed/edited the manuscript. YL wrote the manuscript. MC supervised the study and the guarantor of this work. All authors read and approved the final manuscript.
