# Supplementary material for: Serum microcystin-LR levels and risk of gestational diabetes mellitus: A Chinese nested case-control study
Source: Front Endocrinol (Lausanne). 2023 Jan 4;13:1047866. doi: 10.3389/fendo.2022.1047866 (PMC9846061; doi:10.3389/fendo.2022.1047866)
Supplement: Supplementary file 1 [file Table_1.docx]

**Table S1. Distributions of serum MC-LR concentrations of the total population (N=357)**

|  | <0.1 | 0.10~ | 0.13~ | 0.16~ | 0.19~ | 0.22~ | *P*-value |
| --- | --- | --- | --- | --- | --- | --- | --- |
| Totle | 262(73.4) | 78 (21.8) | 12(3.4) | 1(0.3) | 2(0.6) | 2(0.6) | 0.006 |
| Control | 185(77.7) | 45(18.9) | 8(3.4) | 0 | 0 | 0 |  |
| Case | 77(64.7) | 33(27.7) | 4(3.4) | 1(0.8) | 2(1.7) | 2(1.7) |  |

**Table S2. Distribution of general characteristic by serum MC-LR level in 357 cases**

| Variables | Low (<0.10 ng/ml) | High (0.10-0.23 ng/ml) | *P*-value | |
| --- | --- | --- | --- | --- |
| **Age (years)** |  |  | **0.945** | |
| ≤30 | 150 (57.3) | 54 (56.8) |  | |
| >30 | 112 (42.7) | 41 (43.2) |  | |
| **Pre-pregnancy BMI (kg/m^2^)** |  |  | **0.952** | |
| <24 | 235 (89.7) | 85 (89.5) |  | |
| ≥24 | 27 (10.3) | 5 (10.5) |  | |
| **Educational level** |  |  | **0.559** | |
| High school and below | 35 (13.4) | 15 (15.8) |  | |
| Junior college and above | 227 (86.6) | 80 (84.2) |  | |
| **Occupation** |  |  | **0.086** |  |
| Employed | 225 (85.9) | 89 (92.6) |  | |
| Unemployed | 37 (14.1) | 7 (7.4) |  | |
| **Household income, RMB/month** |  |  | **0.613** | |
| <10000 | 154 (58.8) | 53 (55.8) |  | |
| ≥10000 | 108 (41.2) | 42 (44.2) |  | |
| **Family history of diabetes** |  |  | **0.203** | |
| No | 236 (90.1) | 81 (85.3) |  | |
| Yes | 26 (9.9) | 14 (14.7) |  | |
| **Parity** |  |  | **0.709** | |
| 0 | 157 (59.9) | 59 (62.1) |  | |
| ≥1 | 105 (40.1) | 36 (37.9) |  | |
| **Passive smoking** |  |  | **0.107** | |
| No | 223 (85.1) | 74 (77.9) |  | |
| Yes | 39 (14.9) | 21 (22.1) |  | |
